# Study on the correlation between B vitamins and breast cancer

**DOI:** 10.1186/s12935-023-02860-7

**Published:** 2023-02-09

**Authors:** Siqi Xie, Mingjian Tan, Hongwan Li, Lv Li, Hengyu Zhang, Qing Wang, Sijia Li, Jiali Yang, Haoling Xie, Pengyan Chen, Dequan Liu, Rong Guo, Shicong Tang

**Affiliations:** 1 Department of Breast Surgery, Yunnan Cancer Hospital, The Third Affiliated Hospital of Kunming Medical University, Kunming, Yunnan Province People’s Republic of China; 2 Institute of Oncology, Yunnan Cancer Hospital, The Third Afliated Hospital of Kunming Medical University, Kunming, Yunnan Province People’s Republic of China; 3 Department of Oncology, Anning First People’s Hospital, Kunming, Yunnan Province China; 4 Department of gynecology, Kaiyuan People’s Hospital, Kaiyuan, Yunnan Province China

**Keywords:** B vitamins, Breast cancer, Benign breast diseases

## Abstract

**Background:**

Relevant studies suggest that serum vitamin level is related to the risk of breast cancer, and dietary pattern and drug supplementation can significantly affect the level of vitamin in the body. Therefore, intervention of vitamin level in the body is expected to be a potential strategy to reduce the risk of breast cancer. However, the current epidemiological findings of serum vitamin levels and breast cancer risk are inconsistent, and the relationship between serum vitamin and breast cancer is still controversial. In this study, we compared the serum vitamin expression levels of healthy people, benign breast patients, and breast cancer patients, and evaluated the relationship between B vitamin levels and breast cancer risk.

**Methods:**

The study used liquid chromatography-tandem mass spectrometry to determine the serum vitamin levels of 520 people who attended Yunnan Cancer Hospital from September 2020 to December 2020. After screening by exclusion criteria, 38 patients with benign breast diseases, 87 patients with breast cancer and 91 healthy controls were finally included. The kruskal–wallis H test was used to compare the differences in serum vitamin levels of subjects. Χ^2^ test was used to evaluate the relationship between B vitamin level and age,BMI,TNM staging,Ki-67,Her-2,surgery and chemotherapy, and other baseline characteristics and through binary logistic regression analysis, calculating odds ratio and 95% confidence interval (CI) to evaluate the relationship between B vitamins and breast cancer risk.

**Conclusion:**

The levels of VitB1 and VitB5 in the serum of breast cancer patients and patients with benign breast diseases were higher than those in the healthy control group, while the expression levels of VitB3 in breast cancer patients were lower than those in the healthy control group and the breast benign disease groups. The level of VitB1 was positively correlated with breast cancer risk. The VitB3 level was negatively correlated with breast cancer risk. The VitB5 level is not significantly related to the risk of breast cancer.

## Introduction

Breast cancer is a major public issue in the current society, and its incidence is rising in most countries [1, 2]. According to the latest global cancer burden data released by the International Agency for Cancer of the World Health Organization (IARC), the number of new cancer cases worldwide will reach 19.3 million in 2020. Among them, breast cancer will surpass lung cancer, accounting for about 11.7, making it the most common cancer in the world. It is also the second leading cause of cancer deaths in women [3]. Breast cancer reduces the patient's quality of life, shortens their survival time, and also seriously affects the patient’s mental health [4, 5]. With the improvement of surgical methods and the continuous optimization of comprehensive treatments such as chemotherapy, targeted therapy, endocrine therapy and radiotherapy, the prognosis of patients has been further improved, but patients still face the risk of recurrence and metastasis. Therefore, further exploration of more modifiable risk factors, treatment targets and prognostic factors related to breast cancer is essential to reduce the risk of breast cancer and improve the prognosis of patients.

Nutrients are substances that need to be ingested from the external environment in order to maintain all life activities and processes such as reproduction, growth and survival of the body. The nutrients required by the human body mainly include carbohydrates, fats, proteins, vitamins, water and minerals. According to the different needs of the human body, they are divided into macronutrients with greater demand and micronutrients with smaller demand. Macro elements include carbohydrates, fats, proteins and water. Micronutrients include minerals (such as iron, iodine, copper, selenium, etc.) and vitamins. The content of trace elements in the human body is 0.01–0.005% lower than that of the human body. Although its content is extremely small in the human body, it participates in various metabolic processes of the human body and is of great significance for maintaining the normal activities of the body. Its level is lower than or exceeds the normal range and is closely related to anemia, hyperthyroidism, polycystic ovary syndrome, diabetes, chronic degenerative diseases, cancer and other diseases [6–8].

Vitamins are divided into fat-soluble vitamins and water-soluble vitamins according to their solubility properties. Fat-soluble vitamins mainly include vitamin A (retinol), vitamin D (calciferol), vitamin E (tocopherol) and vitamin K (coagulation vitamin); water-soluble vitamins mainly include B vitamins and vitamin C (L-ascorbic acid). Although the content of vitamins in the body is very small, they are indispensable for maintaining and regulating the normal metabolism of the body. Among them, vitamin A (VitA) deficiency can cause growth retardation, night blindness, dry eye and other diseases [9, 10]. Long-term lack of vitamin C (VitC) can lead to systemic microvascular diseases such as scurvy and arteriosclerosis [11, 12]. The lack of serum vitamin D (VitD) is related to various diseases such as rickets, diabetes, and asthma [13–15]. Vitamin E (VitE) can prevent miscarriage and cardiovascular disease [16, 17]. Lack of vitamin K (VitK) can cause coagulopathy and increase the risk of death from cardiovascular disease [18, 19].

In addition to the above diseases, vitamins are also associated with a variety of malignant tumors. Studies have shown that VitC and VitE can reduce the risk of pancreatic ductal cancer [20]; VitC can also inhibit the proliferation of thyroid cancer cells [21]. The high level of VitD in the body is significantly related to the high risk of skin cancer, pancreatic cancer, colorectal cancer, prostate and lymphoma, and is related to the lower incidence of lung cancer and liver cancer [22–25]. However, studies have suggested that VitD supplementation significantly reduces the mortality rate of cancer, but does not reduce the overall cancer incidence [26]. Appropriate VitE supplementation and maintaining a high level of VitE in the body can reduce the risk of lung cancer and gastrointestinal cancer [27, 28].

Breast cancer is the most common cancer in the world, and its occurrence, development and prognosis prediction are also closely related to multivitamins. Studies have shown that higher VitC intake is significantly related to the low incidence and mortality of breast cancer, and can also reduce the invasion and metastasis of breast cancer [29]. Women with low VitD levels in their bodies have a higher risk of breast cancer. The higher the VitD level, the better the prognosis of breast cancer patients and the lower the risk of death [30, 31]. However, studies have also shown that breast cancer patients with higher VitD levels in the body have a lower survival rate [32].The most effective form of VitE, α-tocopherol succinate (α-TOS), has an anti-tumor effect on breast cancer and can significantly inhibit the lung metastasis of breast cancer cells [33, 34]. VitK intake is positively correlated with the risk of breast cancer [35].

Among vitamins, the B vitamins are a type of water-soluble small molecule compounds. Such compounds do not have similar chemical structures. They are classified as a family due to their many common characteristics, similar biological activities and the need for interaction. It cannot be synthesized in the human body and must be obtained from the outside world. It is widely found in yeast, vegetables, grains, animal liver and so on. The main functions in the human body include vitamin B1 (thiamine), vitamin B2 (riboflavin), vitamin B3 (niacin), vitamin B5 (pantothenic acid), vitamin B6 (pyridoxic acid), vitamin B9 (folic acid) And vitamin B12 (cobalamin). It forms coenzymes and affects the metabolism of carbohydrates, fats, proteins and other substances by participating in redox reactions and hormone signal transduction. Vitamin B2 (VitB2), vitamin B6 (VitB6), vitamin B9 (VitB9) and vitamin B12 (VitB12) are coenzymes of one carbon unit and participate in purine and thymus. Pyrimidine synthesis and homocysteine remethylation are essential for DNA stability and DNA repair. In short, B vitamins are an indispensable substance for the body's metabolism [36, 37].

When the body’s B vitamins are deficient, it can lead to beriberi, angular cheilitis, oral ulcers, peripheral neuritis, megaloblastic anemia and other diseases [38, 39]. Not only that, the level of B vitamins in the serum is also It is related to a variety of cancers and has different roles in the occurrence and development of cancer. Studies have shown that VitB2, VitB9, VitB6 and VitB12 intake can reduce the risk of colorectal cancer through dose–response analysis [40, 41]. With the intake of VitB2, VitB6, folic acid and VitB12 and the increase in body content, the risk of endometrial cancer also increases [42]. Studies have also shown that nicotinamide, a derivative of vitamin B3 (VitB3) in the body, is negatively related to the risk of non-melanoma skin cancer [43]. VitB6 intake can significantly reduce the risk of pancreatic cancer, but the risk of lung cancer is increased [44, 45]. In addition, higher levels of VitB12 in the human body also increase the risk of lung cancer [46]. VitB2, VitB6 and VitB9 can inhibit the proliferation of monocytic lymphoma and exert anti-tumor effects [47].

Similarly, B vitamins are also closely related to breast cancer, but the conclusions on the relationship with breast cancer are inconsistent. Studies have shown that appropriate supplementation of folic acid and VitB12 has a protective effect on BRCA-related breast cancer, especially in BRCA1 mutation carriers [48]. Folic acid in plasma can inhibit the proliferation and metastasis of breast cancer cells, and reduce the risk of recurrence and metastasis of breast cancer [49]. VitB6 Schiff base Mn (II) complex is an effective anti-tumor drug for the treatment of breast cancer [50]. A large intake of VitB2, VitB6, VitB12 and folic acid reduces the overall incidence of breast cancer and the probability of estrogen receptor (ER) positive, progesterone receptor (PR) positive, and Human Epidermal Growth Factor receptor-2 (Her-2) positive subtypes [36]. VitB2, VitB6, and folic acid intake are negatively related to the risk of ER-negative and PR-negative breast cancer, but there is no significant association between VitB12 and breast cancer [37]. However, in a nested case study, it was found that plasma VitB12 compared with the highest quintile and lowest quintile breast cancer, the risk of breast cancer increased by 64% (95% CI 1.17–2.29, P value = 0.02), the higher the level of VitB12 in plasma, the higher the risk of breast cancer. Moreover, VitB9 in plasma is also positively correlated with the risk of invasive breast cancer [51]. Moreover, there is no significant correlation between folic acid and VitB12 in plasma and breast cancer risk and specific breast cancer molecular subtypes [52]. Studies have also pointed out that there is no relationship between the level of B vitamins and the risk of breast cancer [53].

At present, most of the existing studies on the relationship between vitamins and breast cancer risk are conducted in European populations, while there are few studies in Asian populations, and the conclusions of these studies are not consistent. Regarding serum vitamins and breast cancer The conclusion of the relationship between cancers is still controversial, especially the related reports of the role of B vitamins in the occurrence and development of breast cancer are few and the results are mixed. In this study, we plan to analyze the differences in serum vitamin expression levels in healthy people, benign breast diseases, and breast cancer. Explore the correlation between different vitamins and breast cancer tumor stages, ER and PR expression, molecular subtypes, and treatment methods, and evaluate the relationship between B vitamin levels and breast cancer risk.

## Materials and methods

### Research objects

**Fig. 1:**
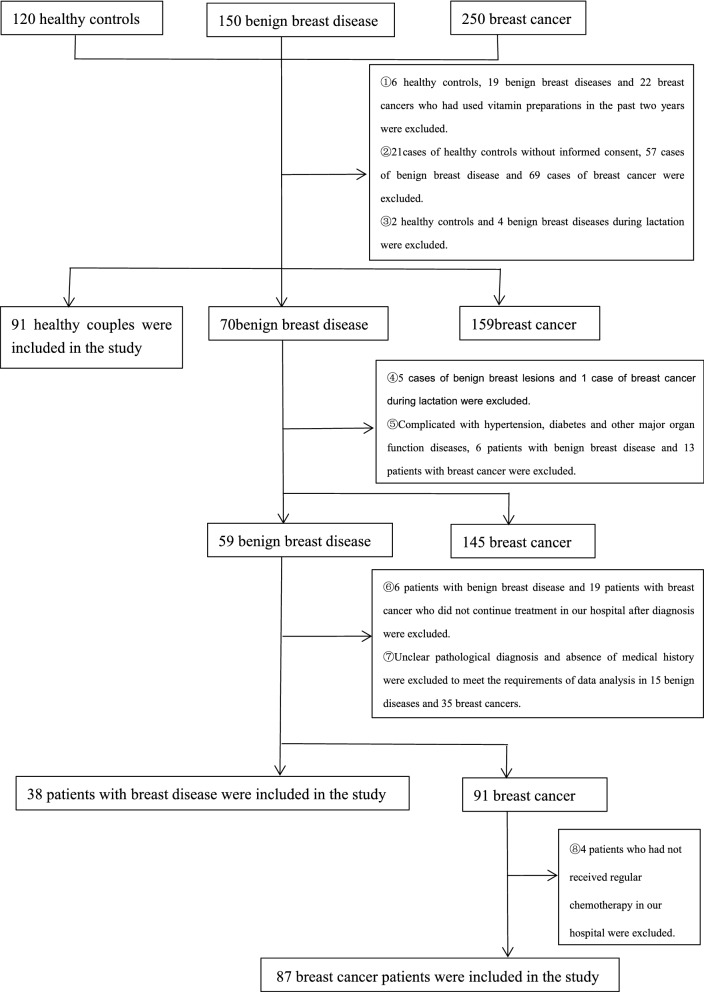
Research object screening flow chart

The study selected 150 patients with benign breast disease (breast benign disease group) and 250 patients with breast cancer (breast cancer group) who were treated at Yunnan Cancer Hospital from September 2020 to December 2020. At the same time, 120 healthy women of the same age group (healthy control group) were measured as a control to compare with the benign tumor group and the breast cancer group. All study subjects were females, aged 23–76 years old, with an average age of 49 ± 1.04 (95%CI 64.94–51.05). After screening by exclusion criteria, 91 cases of healthy control group, 38 cases of benign breast diseases, and 87 cases of breast cancer were finally included (Fig. 1). This study complied with the Declaration of Helsinki (revised in 2013) and was approved by the ethics committee of the Third Affiliated Hospital of Kunming Medical University (Yunnan Cancer Hospital) (No. KY201944). All patients obtained informed consent.

The subjects included in the normal group were healthy women who had no breast-related diseases and met the exclusion criteria, volunteered to cooperate in testing, and provided personal information.

Exclusion criteria: (1) Ingested vitamin preparations within the past two years. (2) Those who have not obtained the love letter. (3) Exclude lactation. (4) Once diagnosed with vitamin deficiency related diseases, such as rickets, beriberi, scurvy, etc. (5) Combined with major organ function diseases such as hypertension and diabetes; (6) After being diagnosed, he did not continue treatment in our hospital to participate in the research. (7) Unclear pathological diagnosis and missing medical history data do not satisfy the data analysis. (8) Breast cancer patients did not receive regular chemotherapy in our hospital.

The subjects have been informed of the contents of the test in the institute, and all subjects have volunteered to participate in the research measurement and signed a written informed consent form.

### Laboratory methods

From September 2020 to December 2020, 520 subjects who were treated at Yunnan Provincial Tumor Hospital from 8 am to the Department of Breast Surgery of Yunnan Provincial Tumor Hospital to collect peripheral venous blood before breakfast on an empty stomach to assess serum vitamin levels. Among them, the blood collection time of the patients in the breast cancer group who are receiving chemotherapy is 8 o'clock in the morning of the second day after the infusion of chemotherapy drugs in the cycle. Each subject collects blood by peripheral venipuncture once and puts it in a coagulation tube, a total of 3-5 ml, and stores it at room temperature. It will be sent to the Cancer Institute of Yunan Tumor Hospital within 1–2 h to detect fat-soluble vitamins and water-soluble vitamins by liquid chromatography-tandem mass spectrometry and issue a test report. The test kits and instruments are all provided by Yun Tumor Hospital Provided by the Cancer Institute.

All subjects’ age, height, weight, menopause, family history of breast cancer and other medical history data were inquired and recorded by the First Tumor and Breast Surgeon in Yunnan Province. Body Mass Index (BMI) is calculated by dividing body weight (kg) by height squared (meters). The pathological data refer to the report obtained by the Department of Pathology of Yunnan Provincial Tumor Hospital.

### Research methods

For patients in the benign tumor group and breast cancer group who had not undergone lumpectomy and pathological examination in the outside hospital, diagnosis and treatment were carried out by two or more breast surgeons from Yunnan Provincial Cancer Hospital. The imaging data obtained from mammography or breast MIR examination, combined with the patient’s medical history and physical examination, assess whether the patient needs breast lumpectomy or breast lump biopsy and histopathological examination. The surgeon performed the main surgery. The excised tissue specimens were diagnosed as benign breast disease or breast cancer by more than 2 physicians from the Department of Pathology of Yunnan Cancer Hospital. In addition, for patients who did not undergo lumpectomy in Yunnan Province and whose pathological specimens of tumors were not preserved in Yunnan Provincial Cancer Hospital, their pathological specimens were all consulted by the Department of Pathology of Yunnan Provincial Cancer Hospital and diagnosed as breast cancer by more than 2 physicians in the Department of Pathology.

All breast cancer patients’ pathological diagnosis, immunohistochemical results, and Fish gene test were obtained by the Department of Pathology, Yunnan Cancer Hospital. The chemotherapy patients in the breast cancer group were all undergoing chemotherapy in the Department of Breast Surgery of Yunnan Cancer Hospital and they were all undergoing chemotherapy. The chemotherapy regimen was determined by the physicians outside the breast of Yunnan Cancer Hospital based on the patient’s pathological examination data and the patient’s personal physical condition Program.

According to the data measured by the Cancer Institute of Yunnan Cancer Hospital, the serum vitamin levels of the healthy control group, the breast benign disease group, and the breast cancer group were compared to analyze their differences. According to the median serum B vitamin levels of breast cancer patients, divide them into different subgroups, analyze the B vitamin levels and age, menstrual status, age at menarche, family history of breast cancer, BMI, TNM staging of breast cancer patients, Tumor size, lymph node status, expression of ER and PR, Ki-67, Her-2, Androgen Receptor (AR), pathological type, surgery, chemotherapy and other baseline characteristics. To assess the relationship between the level of B vitamins and the risk of breast cancer.

### Statistical methods

All data were statistically processed with SPSS 25.0 software, and continuous variables were expressed in median and interquartile range, and P value < 0.05 was considered statistically significant. The measurement data of all vitamin levels in the study were analyzed by KS test and PP diagram. The data showed a non-normal distribution. Therefore, the kruskal–wallis H test was used to perform statistical analysis on the data to evaluate the healthy control group, the breast benign tumor group, and the breast cancer group. Significance of the difference in serum vitamin levels between. The enumeration data used the χ^2^ test, and the minimum expected value was less than 5. The Pearson chi-square test was used to analyze the significance of the differences between the subtypes of breast cancer. Binary logistic regression analysis, calculation of odds ratio and 95% confidence interval (CI) were used to assess the relationship between B vitamins and breast cancer risk.

### Statement of ethics

The author is responsible for all aspects of the work to ensure that issues related to the accuracy and completeness of any part of the work are properly investigated and resolved. Subjects have given their written informed consent and that the study protocol was approved by the institute’s committee on human research.

Study approval statement:This study complies with the Declaration of Helsinki (revised in 2013) and was approved by the ethics committee of the Third Affiliated Hospital of Kunming Medical University (Yunnan Cancer Hospital) (No. KY201944).

Consent to participate statement:Written informed consent was obtained from participants to participate in the study.

## Results

### The serum vitamin concentration of the three groups showed different expression

The study included a total of 216 women, divided into three groups according to breast disease, 91 in the healthy control group, 38 in the benign breast disease group, and 87 in the malignant tumor group.

Table 1 The kruskal–wallis H test was used to analyze whether there are differences in serum levels of various vitamins in the healthy control group, the breast benign disease group, and the breast cancer group. The results show that vitamin D2 (VitD2), VitK and VitE, Vitamin B1 (VitB1), VitB3, Vitamin B5 (VitB5), VitB9, VitC levels are different in the healthy control group, benign disease group, and breast cancer group, and their P value < 0.05, the difference is statistically significant. And VitA, vitamin D3 (VitD3), VitB2, VitB6, vitamin B7 (VitB7), VitB12 P value > 0.05, the difference was not statistically significant.

**Table 1:** Comparison of vitamin concentration levels among healthy controls, benign breast disease patients and breast cancer patients (n = 216)

Variables (n = 216)	Healthy controls (n = 91)		Benign breast disease patients (n = 380)		Breast cancer patients (n = 87)		H	p-value^b^
	Median	Interquartile range	Median	Interquartile range	Median	Interquartile range		
VitA(ng/ml)^a^	405.28	151.37	368.355	166.48	401.37	1488.19	2.212	0.331
VitD2(ng/ml)^a^	1.20	1.07	1.94	3.76	1.45	1.73	10.275	0.006
VitD3(ng/ml)^a^	16.8	5.93	19.71	13.33	18.685	11.78	2.661	0.264
VitK(ng/ml)^a^	0.19	0.20	0.12	0.15	0.17	0.20	6.443	0.040
VitE(ug/ml)^a^	13.38	6.84	7.795	4.79	11.90	7.34	29.964	< 0.001
VitB1(ng/ml)^a^	0.66	0.31	1.335	1.07	1.155	0.94	58.426	< 0.001
VitB2(ng/ml)^a^	4.36	5.31	5.025	7.47	4.575	4.06	2.455	0.293
VitB3(ng/ml)^a^	15.18	16.26	22.44	16.78	13.415	12.68	17.101	< 0..001
VitB5(ng/ml)^a^	23.41	14.39	30.45	13.03	32.455	19.57	15.479	< 0..001
VitB6(ng/ml)^a^	2.10	1.59	2.53	1.35	2.085	1.54	4.683	0.096
VitB7(ng/ml)^a^	0.17	0.46	0.12	0.20	0.17	0.25	4.146	0.126
VitB9(ng/ml)^a^	4.51	3.89	7.55	11.23	4.165	4.05	14.175	0.001
VitB12(ng/ml)^a^	0.02	0.02	0.245	0.03	0.235	0.03	4.258	0.119
VitC(ug/ml)^a^	6.83	3.88	5.465	6.76	4.78	6.49	11.164	0.004

^a^Results are presented in the median (quartile range)

^b^P values tested by kruskal–wallis H test

**Fig. 2:**
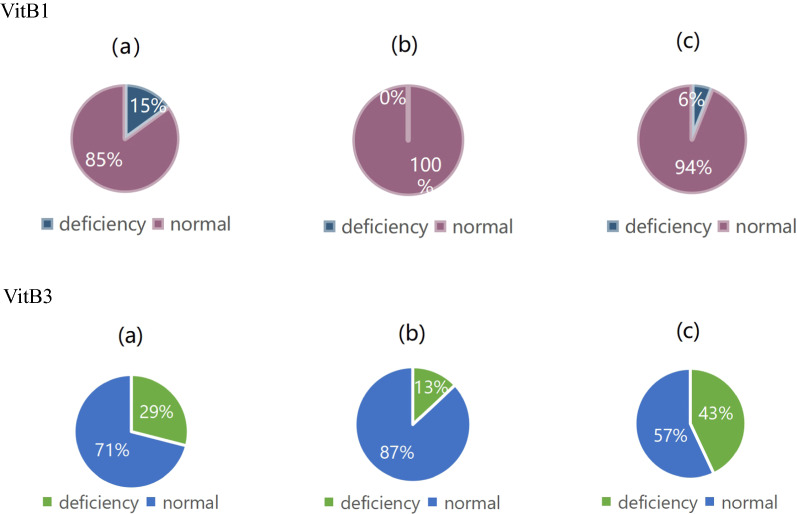
The expression of VitB1 and Vit3 in the three groups of subjects **a** healthy controls, **b** benign breast disease, **c**breast cancer. VitB1 VitB3

The B vitamins VitB1, VitB3, and VitB5 were selected from the vitamins with statistically significant differences in the three groups of study subjects for further analysis. Among the 91 healthy controls, 14 subjects lacked VitB1 and 26 lacked VitB3. In the 38 benign breast tumor group, 5 patients lacked VitB3. In the 87 breast cancer group, 5 patients lacked VitB1 and 37 lacked VitB3. The VitB5 of all study subjects was within the normal range (Fig. 2). Compare the distribution differences of VitB1, VitB3, and VitB5 levels in different groups, and judge that the shape of VitB1, VitB3, and VitB5 levels in each group is basically the same according to the histogram. The distribution of VitB1 (H = 58.426, p < 0.001), VitB3 (H = 17.101, p < 0.001), and VitB5 (H = 15.479, p < 0.001) levels in each group was not all the same, and the difference was statistically significant.

However, the above analysis results can only show that there are differences in the serum concentration levels of VitB1, VitB3, and VitB5 in the three groups of subjects. In order to further analyze the different groups, the Bonferroni method is used to correct the significance level in Table 2 (two-sided test, significance level) A post-hoc comparison of 0.05) found that the level of VitB1 was statistically different between the healthy control group and the benign breast disease group (adjusted P < 0.001), and between the healthy control group and the breast cancer group (adjusted P < 0.001) significance. The levels of VitB3 were statistically significant between the healthy control group and the breast cancer group (P = 0.047 after adjustment), and between the breast benign disease group and the breast cancer group (P < 0.001 after adjustment). The level of VitB5 between the healthy control group and the benign tumor group (P = 0.020 after adjustment), and between the healthy control group and the breast cancer group (P = 0.001 after adjustment), the difference was statistically significant. The data shows that VitB1 levels in breast benign disease groups and breast cancer patients are higher than those in healthy controls; VitB3 levels in breast cancer patients are lower than those in healthy controls and breast benign disease groups; VitB5 levels in breast cancer patients and breast benign disease patients are both high In the healthy control group.

**Table 2:** Pairwise comparison of B vitamin concentrations in Healthy controls, Benign mass and Breast cancer

Variables	VitB1(ng/ml)^a^		*P* value^b^	VitB3(ng/ml)^a^		*P* Value^b^	VitB5(ng/ml)^a^		*P* value^b^
	Median	Interquartile range		Median	Interquartile range		Median	Interquartile range	
Healthy controls	0.66	0.31	< 0.001	15.18	16.26	0.086	23.41	14.39	0.020
Benign mass	1.335	1.07		22.44	16.78		30.45	13.03	
Healthy controls	0.66	0.31	< 0.001	15.18	16.26	0.047	23.41	14.39	0.001
Breast cancer	1.155	0.94		13.415	12.68		32.455	19.57	
Benign mass	1.335	1.07	1.000	22.44	16.78	< 0.001	30.45	13.03	1.000
Breast cancer	1.155	0.94		13.415	12.68		32.455	19.57	

^a^Results are presented in the median (quartile range)

^b^P value of non-parametric data from Kruskal–Wallis H test corrected by Bonferroni method

### Chemotherapy can affect serum VitB3 and VitB5 concentrations in breast cancer patients, but there is no significant difference in serum VitB3 and VitB5 levels in breast cancer patients receiving different chemotherapy regiments

In order to explore whether the levels of VitB1, VitB3, and VitB5 in breast cancer patients are different in baseline characteristics, for the 87 breast cancer patients included in the study, Table 3 is based on the median serum levels of VitB1, VitB3, and VitB5 in the breast cancer group. The median of VitB1 is 1.15 ng/ml, the median of VitB3 is 13.5 ng/ml, and the median of VitB5 is 32.47 ng/ml. The χ^2^ test is used to determine the age of breast cancer patients, whether they are menopausal, age at menarche, and whether they have breasts. A series of baseline characteristics of cancer family history, BMI, TNM stage, tumor size, presence or absence of lymph node metastasis, ER and PR expression, Ki-67, Her-2, AR, pathological classification, chemotherapy, and surgery were statistically analyzed,The results show that:

**Table 3:** Baseline characteristics of serum B vitamin levels in breast cancer patients

Characteristic	VitB1(ng/ml)				VitB3 (ng/ml)				VitB5 (ng/ml)			
	<1.15	≥1.15	χ^2^	*P* Value^a^	< 13.5	≥ 13.5	χ^2^	*P* value^a^	< 32.47	≥ 32.47	χ^2^	*P* value^a^
	N = 42	N = 45			N = 43	N = 44			N = 43	N = 44		
Age(year)			0.095	0.336			1.338	0.239			14.075	< 0.001
< 50	23	20			24	19			30	13		
≥ 50	19	25			19	25			13	31		
Menopause			0.005	0.946			0.171	0.679			6.139	0.013
Yes	12	12			11	13			7	17		
No	30	31			31	30			36	25		
Unknown	0	2			1	1			0	2		
Age of menarche(year)			0.355	0.551			0.153	0.696			2.513	0.113
< 14	15	18			18	15			21	12		
≥ 14	22	20			21	21			19	23		
Unknown	5	7			4	8			3	9		
Family history of breast cancer			1.184	0.277			1.131	0.717			1.186	0.276
Yes	2	5			3	4			2	5		
No	40	40			40	40			41	41		
BIM			2.876	0.237			4.261	0.119			1.906	0.386
< 18.5	1	5			2	4			4	2		
≤ 18.5 < 24	25	22			28	19			25	22		
≥ 24	16	18			13	21			14	20		
TNM			2.961	0.228			1.397	0.497			3.703	0.157
I-II	27	29			31	27			31	27		
III	10	10			8	12			6	14		
IV	3	10			1	2			2	1		
Unknown	2	4			3	3			4	2		
Tumor size(cm)			1.355	0.716			2.178	0.536			3.243	0.356
Tx	6	5			4	7			7	4		
≤ 2	16	20			19	17			16	20		
< 2 ≤ 5	16	18			16	18			17	17		
≥ 5	1	1			2	0			2	0		
T4	3	1			2	2			1	3		
LN metastasis			0.127	0.721			2.759	0.108			0.014	0.905
(+)	18	21			23	16			19	20		
(−)	24	24			20	28			24	24		
ER			0.640	0.424			0.186	0.666			0.854	0.355
(+)	33	32			33	32			34	31		
(−)	9	13			10	12			9	13		
PR			0.525	0.469			3.323	0.072			1.474	0.225
(+)	34	39			33	40			34	39		
(−)	8	6			10	4			9	5		
ki-67			2.612	0.106			2.177	0.140			3.778	0.052
< 14%	16	10			16	10			17	9		
≥ 14%	26	35			27	34			26	35		
HER2			0.102	0.749			2.761	0.097			0.774	0.379
( +)	11	12			8	15			9	14		
(−)	29	27			31	25			28	28		
Unknown	2	6			4	4			6	2		
AR			0.092	0.762			1.568	0.211			3.973	0.047
(+)	27	30			26	31			29	28		
(−)	6	8			9	5			3	11		
Unknown	9	7			8	8			11	5		
Pathological type			0.447	0.800			0.322	0.851			3.205	0.201
Invasive ductal carcinoma	35	39			37	37			35	39		
Invasive lobular carcinoma	2	1			1	2			3	0		
Other	5	5			5	5			5	5		
Chemotherapy			0.044	0.834			10.299	0.001			7.512	0.006
Yes	29	32			37	24			36	25		
No	13	13			6	20			7	19		
Surgery			0.223	0.636			7.777	0.005			2.140	0.143
Yes	25	29			33	21			30	24		
No	17	16			10	23			13	20		

*BMI* body mass index, *TNM* tumor stage, *T* primary foci, *N* lymph node, *M* distant metastasis, *Ln* lymph node, *ER* estrogen receptor, *PR* progesterone receptor, *Ki-67* cell proliferation associated antigen, *HER2* human epidermal growth factor receptor-2, *AR* androgen receptor

^a^P values tested by χ^2^

There were 42 cases in the VitB1 (< 1.15 ng/ml) group and 45 cases in the VitB1 (≥ 1.15 ng/ml) group. There was no difference in the baseline characteristics of breast cancer between the two groups. There were 43 cases in the VitB3 (< 13.5 ng/ml) group and 44 cases in the VitB3 (≥ 13.5 ng/ml) group. The results showed whether the two groups were chemotherapy (χ^2^ = 10.299, P = 0.001) and surgery (χ^2^ = 7.777, P = 0.005). The level of VitB3 in the chemotherapy group was lower than that in the non-chemotherapy group, and the level of VitB3 in the surgery group was also lower than that in the non-surgery group. There were no significant differences in age, menopause, menmene age, family history of breast cancer, BM, TNM stage, tumor size, lymph node metastasis, ER and PR expression, Ki-67, Her-2 gene expression, AR and pathological typing..There were 43 cases in the VitB5 (< 32.47 ng/ml) group and 44 cases in the VitB5 (≥ 32.47 ng/ml) group. The results showed that the age between the two groups (χ^2^ = 14.075, P < 0.001), whether menopause (χ^2^ = 8.140) There are differences in P = 0.017), AR (χ^2^ = 6.848, P = 0.033), chemotherapy (χ^2^ = 7.512, P = 0.006), and at menarche age, whether there is a family history of breast cancer, BMI, TNM staging, tumor There were no significant differences in size, presence or absence of lymph node metastasis, expression of ER and PR, Ki-67, Her-2, pathological classification, and whether or not surgery.

In recent years, molecular typing of breast cancer has been performed by detecting the protein expression or gene amplification of ER, PR, Her-2 and Ki-67 in breast cancer tissues by immunohistochemistry (IHC) and fluorescence in situ hybridization (Fish). This provides A theoretical basis for exploring the heterogeneity of breast cancer cells, as well as an important basis for patient prognosis assessment and individualized precision therapy. According to the 2020 edition of CSCO guidelines for breast cancer diagnosis and treatment, positive PR20% is considered as the critical value for Luminal A and Luminal B, Ki-67 < 15% was low expression, > 30% was high expression, 87 breast cancer patients were molecularly classified. After molecular classification, there were 15 cases of Luminal A type, 34 cases of Luminal B type, and 10 cases of triple-negative type. HER-2 10 cases were positive (HR positive), 11 cases were HER-2 positive (HR negative), and the remaining 7 cases were divided into the unknown group due to missing Her-2 results (Cerb-b2,2 +). Table 4 compares the serum levels of VitB1, VitB3 and VitB5 in breast cancer patients in different molecular typing groups. The results show that the serum levels of VitB1, VitB3 and VitB5 are not statistically different in different molecular typing groups.

**Table 4:** Comparison of serum vitamin B levels in breast cancer patients with different molecular types

Subtype	VitB1(ng/ml)				VitB3 (ng/ml)				VitB5 (ng/ml)			
	< 1.15	≥ 1.15	χ^2^	*P* Value^a^	< 13.5	≥ 13.5	χ^2^	*P* Value^a^	< 32.47	≥ 32.47	χ^2^	*P* Value^a^
	N = 42	N = 45			N = 43	N = 44			N = 43	N = 44		
Luminal A	9	6	3.595	0.609	10	5	4.217	0.519	9	6	4.265	0.512
Luminal B	18	16			17	17			16	18		
TNBC	3	7			5	5			5	5		
HER-2 postive (HR postive)	5	5			3	7			5	5		
HER-2postive (HR negative)	5	6			4	7			3	8		
Unknow	2	5			4	3			5	2		

HER2,human epidermal growth factor receptor-2, *TNBC* three negative breast cancer, *HR* hormone receptor

^a^P values tested by χ^2^

The baseline characteristics analysis of breast cancer showed that the serum VitB3 and VitB5 levels of patients in the breast cancer group were different in chemotherapy.Considering the large number of chemotherapy drugs for breast cancer, different chemotherapy regiments may affect the level of serum B vitamins in breast cancer patients. We further analyzed the serum VitB3 and VitB5 concentrations of 62 breast cancer patients in the chemotherapy groupt. Table 5 is grouped according to the median of serum VitB3 and VitB5 concentrations in the breast cancer chemotherapy group. The medians of VitB3 and VitB5 are 10.39 ng/ml and VitB3 ≥ 10.39, respectively. ng/ml analysis of whether there is a difference in the level of each vitamin in the chemotherapy regimen, the results showed: VitB3 < 10.39 ng/ml group and VitB3 ≥ 10.39 ng/ml group, VitB5 < 28.33 ng/ml group and VitB5 ≥ 28.33 ng/ml group. There was no significant difference in vitamin levels in chemotherapy regimens.

**Table 5:** Comparison of serum B vitamins and chemotherapy regimens in breast cancer patients

Variables	VitB3(ng/ml)				VitB5(ng/ml)			
	< 10.39	≥ 10.39	χ^2^	*P* Value^a^	< 28.33	≥ 28.33	χ^2^	*P* Value^a^
	N = 30	N = 31			N = 30	N = 31		
TC	8	3	7.531	0.057	2	9	5.834	0.120
AC/EC	13	9			13	9		
AC-T/EC-T	6	16			11	11		
Other	3	3			4	2		

*Taxanes* + cyclophosphamide, *AC/EC* anthracycline + cyclophosphamide, *AC-T/EC-T* anthracycline + cyclophosphamide sequencedpaclitaxel, *Other* other chemotherapy regimens in addition to the above chemotherapy regimens

^a^P values tested by χ^2^

Similarly, surgical methods for breast cancer vary according to the specific stage of breast cancer, tumor site, auxiliary treatment conditions of medical units, surgical methods used by surgeons and other factors, and different surgical methods may also affect the serum of breast cancer patients. 87 breast cancer patients were grouped according to the surgical method. 33 cases did not undergo surgery, 10 cases underwent breast-conserving surgery, 40 cases underwent total resection, and the remaining 4 cases underwent other surgical methods. Table 6 compares different operations. The concentration levels of VitB1, VitB3 and VitB5 in the serum of breast cancer patients in the method. The results show that: only the serum VitB3 (χ^2^ = 8.311, P = 0.040) has a atatistically significant difference in different surgical methods. The serum levels of VitB1 and VitB5 There was no statistically significant difference in different surgical methods.

**Table 6:** Comparison of serum B vitamin levels in breast cancer patients under different surgical procedures

Variables	VitB1(ng/ml)				VitB3(ng/ml)			VitB5(ng/ml)				
	< 1.15	≥ 1.15	χ^2^	P^a^	< 13.5	≥ 13.5	χ^2^	P^a^	< 32.47	≥ 32.47	χ^2^	P^a^
	N = 42	N = 45			N = 43	N = 44			N = 43	N = 44		
No surgery	17	16	1.328	0.722	10	23	8.311	0.040	13	20	6.274	0.099
BCT	4	6			7	3			4	6		
mammectomy	20	20			24	16			22	18		
Other	1	3			2	2			4	0		

*BC* breast-conserving therapy, mammectomy(include Simple mastectomy,Modified radical mastectomy and Radical mastectomy)

^a^P values tested by χ2

### VitB1 level was positively correlated with breast cancer risk, VitB3 level was negatively correlated with breast cancer risk, while serum VitB5 level was not significantly correlated with breast cancer risk

**Fig. 3:**
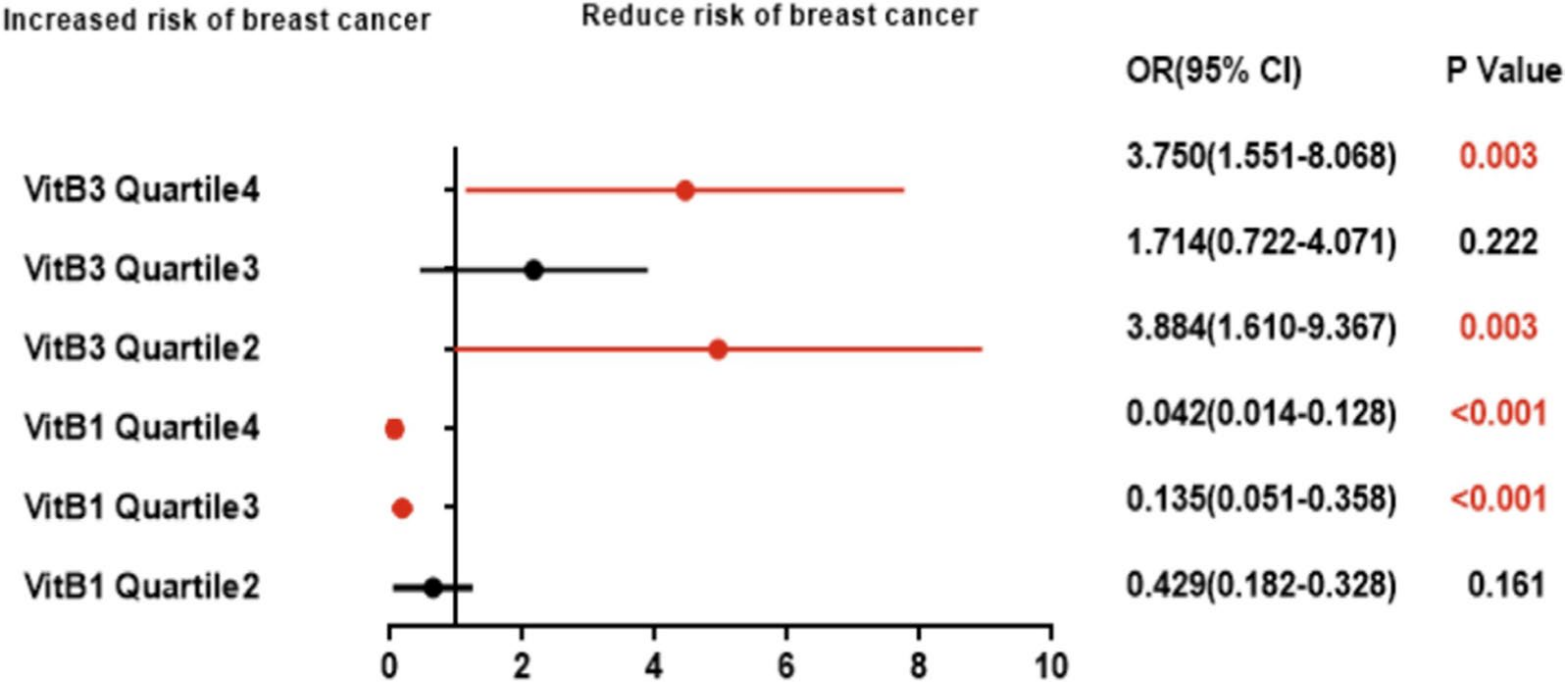
Relationship between VitB1、VitB3 levels and breast cancer risk OR,odds ratio;95% CI, 95% confidence interva

Binary logistic regression analysis, calculation of odds ratio and 95% confidence interval (CI) were used to assess the relationship between B vitamins and breast cancer risk (Fig. 3). The results showed that the level of VitB1 in serum was related to the risk of breast cancer (P < 0.001), and the difference was statistically significant; its regression coefficient B > 0.0 (B = 2.034), indicating that the level of VitB1 was positively correlated with the risk of breast cancer (OR = 7.648, CI 3.279–17.837), that is, the higher the VitB3 content, the higher the risk of breast cancer. The odds ratio between the highest quarter group and the lowest quarter group was 0.042 (Table 7). In addition, the level of VitB3 in serum is also related to the risk of breast cancer (P = 0.006), and the difference is statistically significant; its regression coefficient B < 0.0 (B = − 0.052), indicating that the level of VitB3 is negatively correlated with the risk of breast cancer (OR = 0.950, CI 0.916–0.985), that is, the lower the VitB3 content, the higher the risk of breast cancer. The odds ratio between the highest quarter group and the lowest quarter group was 3.570 (Table 8). The level of VitB5 in serum is not significantly related to the risk of breast cancer.

**Table 7:** Relationship between serum vitamin B1 levels and breast cancer risk

Subjects	Quartile 1(Q1)	Quartile 2 (Q2)	Quartile 3 (Q3)	Quartile 4 (Q4)
Healthy controls (n)^a^	8	14	28	37
Breast cancer (n)^a^	36	31	17	7
OR(95%CI)	Reference	0.429(0.182–1.328)	0.135(0.051–0.358)	0.042(0.014–0.128)
p-value^b^	Reference	0.161	< 0.001	< 0.001

*OR*,odds ratio, 95% *CI* 95% confidence interval

^a^Results are presented as a median (quartile range) hierarchical count

^b^P values derived from binary logistic regression analysis

**Table 8:** Relationship between serum vitamin B3 levels and breast cancer risk

Subjects	Quartile 1(Q1)	Quartile 2 (Q2)	Quartile 3 (Q3)	Quartile 4 (Q4)
Healthy controls(n)^a^	30	16	35	16
Breast cancer (n)^a^	14	29	20	28
OR(95%CI)	Reference	3.884(1.610–9.367)	1.714(0.722–4.071)	3.750(1.551–8.068)
p-values^b^	Reference	0.003	0.222	0.003

*OR*,odds ratio, 95% *CI* 95% confidence interval

^a^Results are presented as a median (quartile range) hierarchical count

^b^P values derived from binary logistic regression analysis

In conclusion, B-group vitamin levels are different in different populations. Serum VitB1 and VitB5 levels in patients with breast cancer and benign breast diseases are higher than those in healthy control group, while the expression level of VitB3 in patients with breast cancer is lower in healthy control group and benign breast disease group. Subgroup analysis also suggested that chemotherapy could affect serum VitB3 and VitB5 concentrations in breast cancer patients, but there was no significant difference in serum VitB3 and VitB5 levels in breast cancer patients receiving different chemotherapy regimings. Our study also showed that VitB1 levels were positively correlated with breast cancer risk and that the odds ratio between the highest and lowest quartile was 0.042. VitB3 level was negatively correlated with breast cancer risk and the odds ratio between the highest and lowest quartile groups was 3.570, while serum VitB5 level was not significantly correlated with breast cancer risk.

## Discussion

In this study, we used liquid chromatography-tandem mass spectrometry to measure the serum levels of fat-soluble vitamins and water-soluble vitamins in women with benign breast diseases, breast cancer patients, and healthy controls. The differences in vitamin levels among patients with benign breast diseases, breast cancer patients and healthy controls were compared. The differences in VitB1, VitB3, and VitB5 between breast cancer patients and healthy people were reported for the first time, and VitB1, VitB3 and VitB5 were discussed. The relationship between Vitamin B level and breast cancer tumor staging, molecular classification, lymph node metastasis, surgical methods and chemotherapy regimens.

As mentioned above, Compared with healthy controls, the expression level of VitC in breast cancer patients was lower (P = 0004), suggesting that low levels of VitC may be related to the occurrence of breast cancer. Therefore, it may be possible to reduce the risk of breast cancer by increasing the level of VitC in the body through additional VitC supplements.Studies have shown that increasing VitC intake can inhibit the proliferation of breast cancer cells in vitro and in vivo by reducing glycolysis and protein synthesis, and can also inhibit cell migration and invasion of breast cancer cell lines by inhibiting epithelal-mesenchymal transition, significantly reducing the incidence and overall mortality of breast cancer [54, 55]. Similarly, Zhang D et al. also proposed that higher VitC intake is significantly related to the low incidence and mortality of breast cancer, and can also reduce the invasion and metastasis of breast cancer. In addition, although many studies have shown that breast cancer patients have lower serum VitD levels, and lower VitD levels are positively correlated with breast cancer risk, there are also studies that have shown that breast cancer patients with higher VitD levels in the body have higher levels of VitD. The survival rate is low, and VitD supplementation does not reduce the risk of breast cancer. In our study, the levels of VitD2 in benign breast tumors and breast cancer were higher than those in the healthy control group, but there was no significant difference in the expression of VitD3 in the three groups of subjects. It follows that VitD or VitD supplementation cannot be used to reduce the risk of breast cancer.

B vitamins are known to participate in the methylation cycle as coenzymes, acting on the synthesis of DNA, RNA, proteins and phospholipids in the body. When the abnormal level of B vitamins in human serum causes any of these metabolic pathways to be interrupted, it may interfere with DNA replication, repair, and regulation of gene expression, and ultimately promote cell cancer [36, 37]. However, there are currently few studies on the relationship between B vitamins and breast cancer, and the role of B vitamins in the occurrence and development of breast cancer is still inconclusive. In our research, we measured the B vitamins that play a major role in the human body and found that VitB2, VitB6, VitB7 and VitB12 have no significant differences in healthy controls, patients with breast benign diseases, and breast cancer patients, which also suggests that VitB2, VitB6, VitB7, VitB12 have no significant correlation with breast cancer risk. Similarly, Zeng J et al. conducted a prospective study and meta-analysis of one carbon unit and breast cancer risk and also proposed that there is no significant association between VitB12 and breast cancer [37]. Related research by Arthur RS et al. also proposed that there is no significant association between group B vitamins and breast cancer [53]. This is consistent with some of our research results.In addition, the different expressions of VitB1, VitB3 and VitB5 in breast cancer patients suggest that the change of their concentrations may be related to the occurrence and development of breast cancer, which provides a certain basis for further exploration of the role of vitamins in the etiology of breast cancer. This also indicates to some extent that monitoring the levels of VitB1, VitB3 and VitB5 in vivo and being alert to the changes of their levels are conducive to the early detection and diagnosis of breast cancer.

Previously, few studies reported the relationship between VitB1, VitB3, VitB5 and breast cancer. Our study suggests that serum VitB3 in breast cancer patients may be affected by chemotherapy and surgery. The serum VitB3 concentration was lower in the chemotherapy group.This may be due to nausea, vomiting and other side effects of chemotherapy that led to a decrease in the intake of VitB3 in food, or insufficient absorption of VitB3 due to diarrhea during chemotherapy. At the same time, another major source of B vitamins is intestinal microbes. Gastrointestinal reactions caused by chemotherapy may also lead to changes in intestinal vitamins, resulting in changes in the concentration of VitB3 in patients undergoing chemotherapy [56]. However, different chemotherapy regimens had little effect on serum VitB3 concentration in breast cancer patients. In addition, the level of VitB3 in patients after surgery is relatively low, which may be due to the dietary changes of patients after surgery and the stress state of the body, leading to the decrease of VitB3 intake and absorption. The different surgical methods will also affect the serum VitB concentration of patients, which may be related to the difference in the scope of surgery, the time required for postoperative recovery and the different effects on postoperative life, resulting in changes in vitamin metabolism of the body.

VitB5 concentration level at age < 50 years old group and ≥ 50 years old group (P < 0.001), pre-menopausal group and post-menopausal group (P = 0.017), AR positive group and AR negative group (P = 0.033), and no chemotherapy group and chemotherapy group (P = 0.006) had different expression.. It is suggested that the age, menstrual status, AR expression and whether chemotherapy of breast cancer patients are related to the expression level of vitamin B5. The results suggest that the level of VitB5 in the ≥ 50-year-old group is higher than that in the < 50-year-old group, which may be related to changes in dietary habits, changes in vitamin physiological requirements and the body's ability to absorb vitamins with age. In addition, the levels of VitB5 in the non-menopausal group were lower than those in the menopausal group. This is related to the changes in hormone levels in the premenopausal menstrual cycle and the shedding of the endometrium that affect the absorption and metabolism of VitB5, but its specific impact mechanism needs further study.

Studies have shown that higher serum B vitamins can reduce the risk of breast cancer [38, 39], and some studies support that the higher the serum B vitamin level, the higher the risk of breast cancer [51]; There are also studies that indicate that there is no relationship between B vitamins and breast cancer risk [52, 53]. In this study, we evaluated the relationship between VitB1, VitB3, and VitB, which have different expressions in healthy controls, patients with breast benign diseases, and breast cancer patients, and breast cancer risk, and found that VitB1 is positively correlated with breast cancer risk (OR = 7.648, CI 3.279–17.837), VitB3 content was negatively correlated with breast cancer risk (OR = 0.950, CI 0.916–0.985), while VitB5 levels in the serum were not significantly correlated with breast cancer risk. The vitamin level in human body can be changed through dietary intake and drug supplementation, which also suggests that lowering VitB1 level or increasing VitB3 level in human body may provide new means and methods for clinical prevention and treatment of breast cancer.

The study analyzed differences in the expression of vitamins in different populations, and also assessed the relationship between group B vitamins and breast cancer risk. Prior to this, there were few studies on the relationship between B vitamins and breast cancer, and related studies in Asian populations were also relatively lacking. Our research provides basic results for future research to further prove the role of B vitamins in the occurrence and development of breast cancer.

Our research results also have certain limitations. Although we have excluded some confounding factors that affect vitamin levels in the body, such as the intake of drug vitamins, vitamin deficiency related diseases, etc., the impact of dietary patterns on vitamin levels has not been fully considered. To Secondly, the study included fewer subjects and missing some data. The results may cause errors and cannot provide a comprehensive explanation for the relationship between B vitamins and breast cancer. Therefore, more careful design, more complete data, and larger sample sizes are needed in the future. Many studies have further explored the relationship between serum vitamin levels, the use of vitamin supplements and breast cancer risk and prognosis.

## Conclusion

The levels of VitB1 and VitB5 in the serum of breast cancer patients and patients with benign breast diseases were higher than those in the healthy control group, while the expression levels of VitB3 in breast cancer patients were lower than those in the healthy control group and the breast benign disease groups. The level of VitB1 was positively correlated with breast cancer risk. The odds ratio between the highest quartile and the lowest quartile was 0.042. The VitB3 level was negatively correlated with breast cancer risk. The odds ratio of the highest quarter group to the lowest quarter group was 3.570. The level of VitB5 in serum is not significantly related to the risk of breast cancer.

## Data Availability

The datasets used during the current study are available from the corresponding author on reasonable request.
